# Genes and cells associated with sublingual allergen immunotherapy revealed by integrated transcriptome analysis in allergic rhinitis patients

**DOI:** 10.3389/falgy.2026.1855863

**Published:** 2026-09-03

**Authors:** Zhengqi Li, Yilin Hou, Hang Li, Ding Ji, Yi Wei, Renqiang Ma

**Affiliations:** 1 Department of Otorhinolaryngology, The First Affiliated Hospital of Sun Yat-sen University, Guangzhou, China; 2 Guangzhou Key Laboratory of Otorhinolaryngology, The First Affiliated Hospital of Sun Yat-sen University, Guangzhou, China; 3 Department of Otorhinolaryngology, The Sixth Affiliated Hospital, Sun Yat-sen University, Guangzhou, China; 4 Department of Otorhinolaryngology, Peking University Shenzhen Hospital, Shenzhen, China; 5 Shenzhen Peking University-The Hong Kong University of Science and Technology Medical Center, Shenzhen, Guangdong, China

**Keywords:** allergen immunotherapy, allergic rhinitis, efficacy, natural killer cells, RNA-Seq

## Abstract

**Background:**

Sublingual allergen immunotherapy (SLIT) is known as an effective therapy for allergic rhinitis (AR), although its efficacy varies across different treatment duration and individuals. The factors associated with the duration and response of SLIT need to be explored.

**Methods:**

Twenty-seven AR patients undergoing SLIT and three healthy volunteers were recruited. Peripheral blood mononuclear cells were isolated for RNA sequencing, and key results were further examined by quantitative PCR (qPCR) in available PBMC samples. Time-correlated and response-associated genes were identified and analyzed using bioinformatic tools. The data were further integrated with single-cell transcriptome.

**Results:**

We first identified 164 genes significantly correlated with SLIT duration. The expressions of genes associated with immunoregulation, nitric oxide and serotonin transportation were regulated as the treatment proceeded. qPCR validation further showed a significant positive correlation between SLC6A4 expression and SLIT duration, while SH2D1B and ARG1 showed positive trends. Then, we identified 257 up-regulated and 349 down-regulated genes in SLIT responders. Several pathways were enriched in these genes, including interferon signaling (up-regulated in responders) and granulocytes migration (down-regulated in responders). Single-cell and deconvolution analyses suggested that natural killer cell-associated signals may associate with SLIT efficacy, potentially in relation to T-cell-regulatory and eosinophil-migration-related transcriptional programs.

**Conclusions:**

This study revealed potential genes and cells associated with SLIT duration and response. Regulation of T cells and NK cells may play a crucial role in the successful treatment responses. Further studies are required to validate these findings and assess their clinical relevance.

## Introduction

Allergic rhinitis (AR) is an inflammatory disease of the nasal mucosa, caused by immunoglobulin (Ig)E-mediated histamine release in atopic individuals following exposure to certain allergen (1). The prevalence of AR has been steadily increasing over the last few decades, and currently ranges from 10% to 40% globally (2, 3). The only known disease-modifying therapy for AR is allergen immunotherapy (AIT), which aims to induce immune tolerance through the graduated administration of the allergen extract (4). The efficacy and safety of AIT, and particularly of sublingual immunotherapy (SLIT), have been documented; however, only some of the patients actually benefit from the treatment (5–7). Considering that the lengthy process would place a heavy financial and mental burden on AR patients, it is important to understand the mechanisms affecting the SLIT efficacy which may help to optimize clinical decision (8).

The successful induction of immune tolerance involves multiple factors (9). Firstly, it is known that an adequate duration of treatment is crucial for a positive response to SLIT. Although there is still debate, some studies have indicated that a minimum of 6 months of SLIT is necessary before observable clinical effect (10). However, there has been relatively limited research about the biological factors that might change during the treatment. Additionally, the pivotal biological responses distinguishing SLIT responders from non-responders remain understudied. Research has highlighted some biological changes induced by SLIT. Huoman et al. reported that SLIT adjusted the equilibrium of T helper (Th)1/Th2 cytokines, leading to the suppression of the Th2 response and the activation of the Th1 response (11). Other studies indicated that SLIT-induced immune cells, such as regulatory T cells (Tregs), regulatory B cells (Bregs), and regulatory dendritic cells (DCregs), modulated the allergy response through regulatory signals [e.g., interleukin (IL)-10] (12).

High-throughput sequencing, complemented by single-cell transcriptomics, allows preliminary screening of a vast array of biological factors and identification of highly-involved cell types. In this retrospective study, we employ integrated analysis to investigate genes, pathways, and cells associated with SLIT response, focusing on their correlation with treatment duration and their disparities between responders and non-responders.

## Materials and methods

### Study design and participants

**Figure 1:**
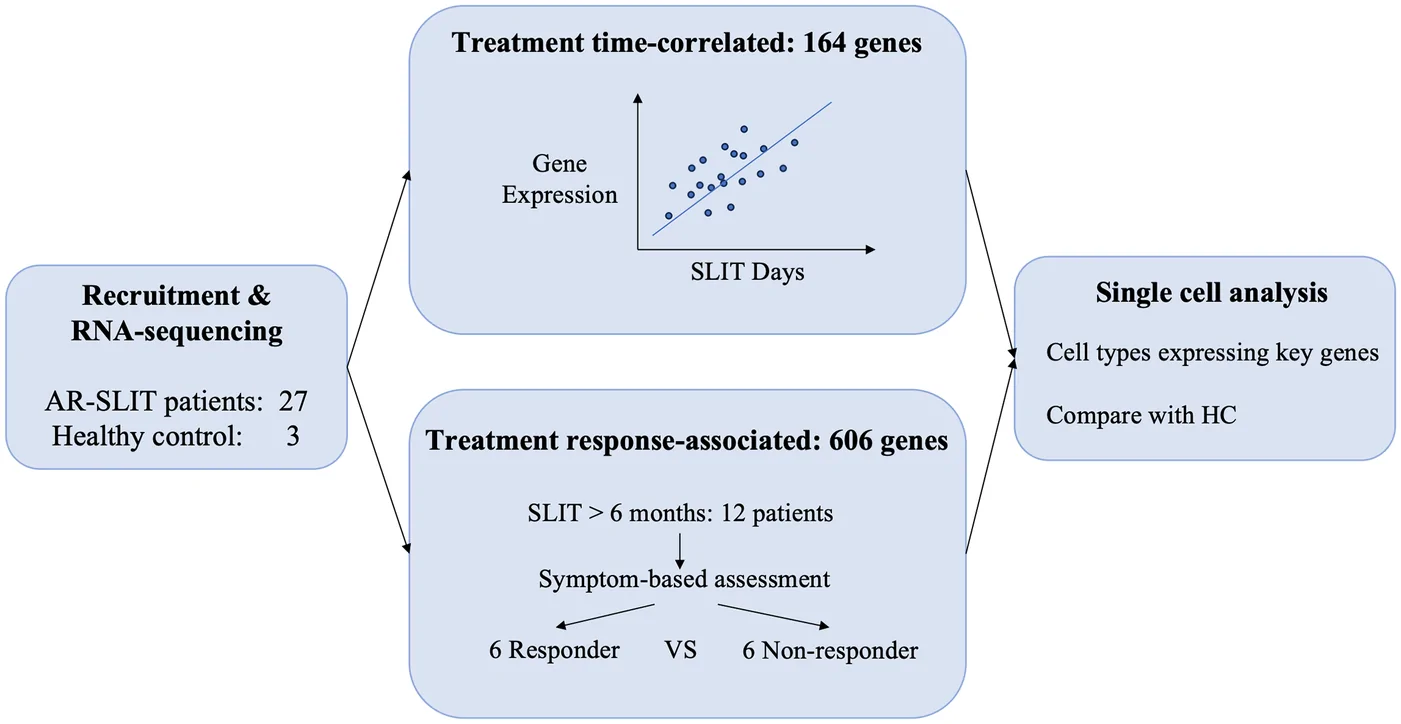
Flowchart of the study design and analysis. Twenty-seven SLIT-treated AR patients and three healthy controls were recruited for RNA sequencing. The overall SLIT cohort was used for the duration-related analysis, whereas the response-analysis subset comprised 12 patients treated for longer than 6 months, including 6 responders and 6 non-responders.

Twenty-seven AR patients who were currently receiving SLIT and three healthy controls (HC) were recruited in the outpatient clinic. The clinical characteristics of the participants and the analysis protocol are summarized in Table 1 and Figure 1. Although all patients were recruited during the same period, they had started SLIT at different times and therefore had different treatment durations at sampling, making this cross-sectional design suitable for correlation analysis between gene expression and treatment time. One patient had an exceptionally long treatment duration (2,178 days) and was excluded from the correlation analysis as an outlier (see the relevant Materials and Methods section). Because the effect of SLIT is generally expected after at least 6 months of treatment (10), the remaining 12 patients who had received SLIT for longer than 6 months were entered into the therapeutic-response analysis and classified as 6 responders and 6 non-responders (Figure 1 and Table 2).

**Table 1:** The demographic and clinical variables of participants.

Variable	SLIT	HC	*P*-value*
n	27	3	-
Sex (Male %)	17 (63.0)	2 (66.7)	0.90
Age [mean (SD) year]	19.81 (10.64)	24.67 (2.08)	0.45
VAS [mean (SD)]	12.15 (6.33)	0.00 (0.00)	<0.01
Treatment duration [mean (SD) day]	336.11 (419.78)	0.00 (0.00)	0.19

SLIT, sublingual allergen immunotherapy; HC, healthy control; VAS, visual analogue scale; SD, standard deviation.

* Assessed using the Fisher exact test or Student's *t*-test.

**Table 2:** The demographic and clinical variables of the 12 patients included in the response analysis.

Variable	Responder	Non-responder	*P*-value*
n	6	6	-
Sex (Male %)	4 (66.67%)	3 (50%)	>0.99
Age [mean (SD) year]	23 (13.84)	20.5 (10.97)	0.76
VAS [mean (SD)]	9.67 (3.94)	18 (5.26)	<0.05
Treat time [mean (SD) day]	740.8 (652.5)	432.2 (227.4)	0.34

VAS, visual analogue scale; SD, standard deviation.

* Assessed using the Fisher exact test or Student's *t*-test.

AR was diagnosed as sensitive to house dust mites based on the standard of Allergic Rhinitis and Its Impact on Asthma (ARIA) guidelines (13), which combine symptom evaluation and the levels of IgE specific to Der p1 (s-IgE; measured using for specific IgE, HOB Biotech Group, Suzhou, China). In this real-world cohort, nasal provocation testing was not routinely performed; accordingly, patient inclusion was based on clinical assessment, Der p1 sensitization, and the treatment context of Df-SLIT. During routine SLIT, all patients were seen by the allergist for symptom evaluation. All the enrolled AR patients had consented to SLIT as part of their allergy treatment, but were excluded if they: (1) had taken anti-inflammatory or anti-allergy drugs (e.g., glucocorticoids) within the last 2 weeks before sampling; (2) had chronic respiratory infection or experienced acute respiratory infection within 2 weeks of sampling; (3) had previously been diagnosed with an immunodeficiency or another systemic immune disorder; or (4) were pregnant. Informed consent was obtained prior to the collection of biological samples and clinical data. All related protocols were approved by the ethics committee (Approval No. 2016197).

### SLIT

In this study, all patients undergoing SLIT were treated with *Dermatophagoides farinae* (*Df*) drops (Changdi, Zhejiang Wolwo Bio-pharmaceutical Co., Ltd., China), according to the manufacturer's instructions. The treatment period consisted of a dose-escalation phase and a maintenance phase. For patients over 14 years of age, the specific administration regimen was as follows: *Df* drops No. 1 (1 µg/mL), No. 2 (10 µg/mL), and No. 3 (100 µg/mL) were administered at weeks 1, 2, and 3, respectively; one, two, three, four, six, eight, and ten *Df* drops were administered on days 1, 2, 3, 4, 5, 6, and 7, respectively. *Df* drop No. 4 (333 µg/mL; three drops each time) was administered during weeks 4 and 5. From week 6 and until the end of the SLIT course, *Df* drop No. 5 (1,000 µg/mL; two drops each time) was administered. In children under 14 years old, the method and dosage for the first 3 weeks of SLIT were the same as for those aged over 14 years. However, from week 4 until the end of the SLIT course, *Df* drop No. 4 (333 µg/mL; three drops each time) was administered. After the initial hospital administration of *Df* drops, the rest of the treatment was self-administered by the patient once a day at home. Each *Df* drop was placed under the tongue and swallowed after 1–3 min. Daily dosing at a fixed time was recommended.

All the patients attended baseline and follow-up visits, where detailed information (including basic information, signs and symptoms, medical history, allergen test results, and medication use) was collected from the patients' records. Regular follow-up visits were scheduled. During treatment, if the patient had a systemic or local allergic reaction, symptoms could be alleviated by adjusting the dose of *Df* drops and/or using antihistamines under the guidance of the clinician.

### Therapeutic effect evaluation

At follow-up visits, the 12 patients included in the therapeutic-response analysis were evaluated using the visual analog scale (VAS) and a subjective questionnaire. Since no reliable biomarkers for SLIT efficacy have been established, some published symptom-based methods have been used to evaluate SLIT effectiveness (14). In this study, we adopted efficacy evaluation methods based on VAS and subjective assessments. Specifically, patients who had a significant reduction (a total reduction ≥ 5 or 30% from baseline) in their VAS scores and subjective relief of their AR symptoms were defined as responders, whereas the remaining patients, including those with no improvement or worsening symptoms, were defined as non-responders. This yielded 6 responders and 6 non-responders, and the demographic variables of these participants are listed in Table 2.

### Sample preparation, RNA sequencing (RNA-Seq) and PCR validation

Peripheral blood mononuclear cells (PBMCs) were isolated from the blood samples of AR patients and HCs using Ficoll-Hypaque density gradient centrifugation in Lymphocyte Separation Medium (MP Biomedicals, Santa Ana, USA). The RNeasy Mini Kit (Qiagen, Germany) was used to extract and purify total RNA. Paired-end libraries were constructed using the TruSeq RNA Sample Preparation Kit (Illumina, USA) following the user guide. Briefly, the poly-A-containing mRNA molecules were purified using poly-T oligo-attached magnetic beads. This mRNA was then fragmented and the cleaved fragments were reverse transcribed into cDNA. After second strand synthesis, end repair, and adapter ligation, the DNA products were purified and enriched using PCR to generate the ready-to-sequence library. Libraries were then quantified and validated on the Qubit® 2.0 Fluorometer (Life Technologies, USA) and the Agilent 2100 bioanalyzer (Agilent Technologies, USA), and sequenced on the Illumina HiSeq 2,500 platform (Illumina, USA). Selected duration-associated genes (SH2D1B, ARG1, and SLC6A4) were further examined by qPCR, using GAPDH as the internal control. Relative expression was calculated as 2^-*Δ*Ct after qPCR quality control and manual review of sample reliability. The primer sequences for SH2D1B, ARG1, SLC6A4, and GAPDH are provided in Supplementary Table S8. The datasets generated and/or analyzed in the present study are available in the Sequence Read Archive repository; https://www.ncbi.nlm.nih.gov/sra/PRJNA808860.

### Computational methods

#### Data preprocessing and expression analysis

Raw data were preprocessed by filtering out low-quality reads. Hisat2 and featureCounts were used to map the cleaned reads to the human hg38 reference genome and to generate a raw count matrix (15, 16). Subsequent analysis was performed using R.

#### Correlation and co-expression network analysis

The Pearson coefficient was used to evaluate correlations between normalized gene expression and SLIT duration (days). Notably, one patient had an extended treatment duration (2,178 days). This patient was excluded from the correlation analysis to ensure results were not biased by outliers. The MEGENA package in R was utilized to construct a gene co-expression network, while Cytoscape was used to construct the network plot (17, 18). The full list of significantly correlated genes can be found in Supplementary Table S1.

#### Differential expression analysis

Differentially expressed features were identified using edgeR (19). The differentially expressed genes (DEGs) were filtered out using the following criteria: FDR ≤ 0.05 and fold-change ≥ 1.5. The Volcano plot was generated using ggplot2. The full list of DEGs can be found in Supplementary Table S2. The overlap genes between correlated genes and DEGs were listed in Supplementary Table S3.

#### Pathway enrichment analyses

The over-representation analysis was performed using MetaScape (20). A custom analysis was run on a default setting. Briefly, enrichment analysis was carried out using variable ontology sources. The expression of all the genes in the genome was used as the enrichment background. Terms with a *P*-value < 0.01, a minimum count of three, and an enrichment factor > 1.5 were grouped into clusters based on their membership similarities. Top pathways were re-plotted using ggplot2. Module scoring was evaluated by gene set variation analysis (GSVA) (21).

The full list of significantly enriched pathways for correlated genes and DEGs can be found in Supplementary Tables S4 and S5, respectively.

#### Single-cell RNA-Seq analysis

A public dataset generated from human PBMCs was acquired and analyzed using Seurat (22). Because this reference dataset was derived from healthy PBMCs rather than disease-matched AR or SLIT samples, it was used to support cell-type annotation and exploratory deconvolution, and may not fully capture disease-specific transcriptional states. The DotPlot function was used to visualize gene expression across cell clusters, the AddModuleScore function was used to calculate gene set scores for each cell, and the FeaturePlot function was used to display gene set scores on a reduced dimensional plot. BayesPrism, a Bayesian framework for the deconvolution of single-cell data, was used to infer cell type proportions and cell-type-specific gene expression profiles (23). edgeR was then used to identify DEGs between each cell type based on the deconvolved expression matrix. The full list of cell-type-specific DEGs can be found in Supplementary Table S6. The full list of natural killer (NK)-cell-specific DEGs derived from the comparison of responders, non-responders, and HCs can be found in Supplementary Table S7.

### Statistical analysis

The specific statistical methods for each analysis are described in the figure or figure legend. Statistical analysis and plot generation were performed using R. Correlations between qPCR relative expression and SLIT duration were assessed by Spearman correlation.

## Results

### Hub genes and pathways related to SLIT duration

**Figure 2:**
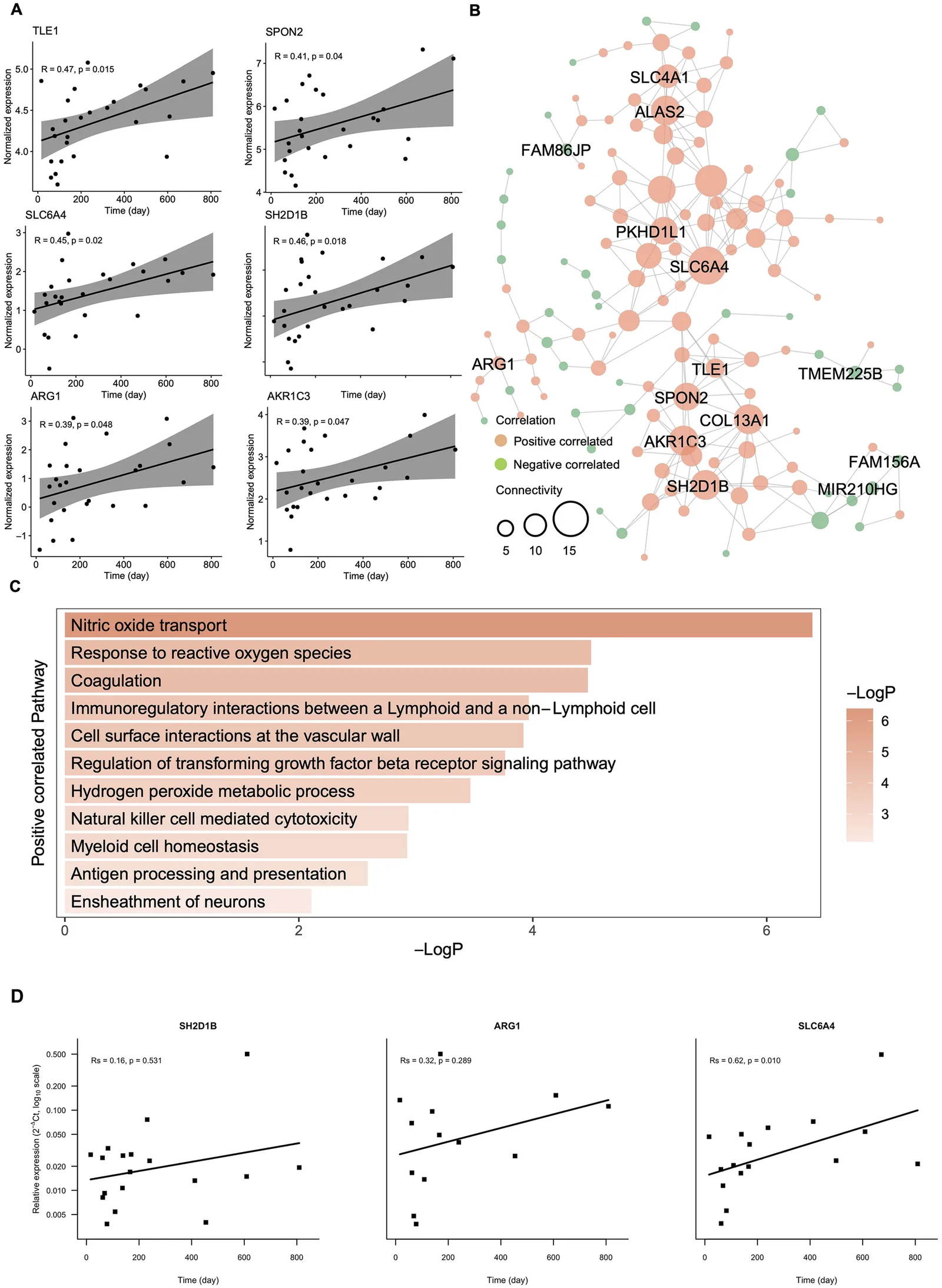
Genes associated with SLIT duration **(A)** scatter plot showing the correlations between the expression of representative genes and the duration of sublingual immunotherapy (SLIT), as determined by Pearson correlation analysis. The lines and shaded areas indicate the linear regression and the 95% confidence interval. **(B)** Network diagram of the time-correlated genes’ co-expression analysis; the nodes represent genes and the links represent the correlation of expression between each pair of genes. The hub genes are labeled. **(C)** The top enriched pathways of 108 genes positively correlated with treatment duration. **(D)** qPCR validation of SH2D1B, ARG1, and SLC6A4 in available PBMC samples. Only samples retained after qPCR quality control and manual review were included. Relative expression was normalized to GAPDH and calculated as 2^-DeltaCt. Associations with SLIT duration were assessed by Spearman correlation. SLC6A4 showed a significant positive correlation, whereas SH2D1B and ARG1 showed positive but non-significant trends. Filled black squares indicate the retained qPCR samples, and the black line indicates the fitted linear regression.

We first assessed the correlation between gene expression and the duration of SLIT. After preliminary feature selection, we correlated gene expression with treatment time and identified 164 significantly associated genes, of which 108 genes were positively and 56 genes were negatively associated with SLIT duration (Figure 2A). Co-expression network was used to identify hubs among these 164 genes (Figure 2B), and pathway enrichment analysis was performed to depict the functional pathways enriched by the 108 positively corelated genes respectively (Figure 2C).

We identified the hub genes *SH2D1B* and *AKR1C3* and the corresponding pathway “antigen processing and presentation” (hsa04612; *P* < 0.01), indicating that the interaction between T cells and antigen presenting cells was increased as the treatment proceeded. Further, genes *ARG1* and *TLE1* were also identified as hub gene*s*, annotated as the members of the “immunoregulatory interactions between a lymphoid and a non-lymphoid cell” pathway (R-HSA-198933; *P* < 0.01). Since *ARG1* and *TLE1* are reported to express on DCs and monocytes, these results implied that longer SLIT treatment tends to induce an antigen-presenting-cells-conducted suppression of T-cell functions. We also found evidence of the modulation of innate immune response, including the “natural killer cell mediated cytotoxicity” (hsa04650; *P* < 0.01), “myeloid cell homeostasis” (GO:0002262; *P* < 0.01), and “response to reactive oxygen species” (GO:0000302; *P* < 0.01) pathways.

Interestingly, the expression of SLC6A4 [encoding the 5-hydroxytryptamine (5-HT; serotonin) transporter enzyme, which inhibits the function of serotonin via transmembrane transportation] was also positively associated with SLIT duration, which may contribute to the long-term symptom remission related to the treatment. We further performed qPCR for SH2D1B, ARG1, and SLC6A4. SLC6A4 showed a significant positive correlation with SLIT duration, while SH2D1B and ARG1 also showed positive trends (Figure 2D). Together, these results provide supportive evidence for the reliability of the duration-associated transcriptomic findings.

### Hub genes associated with the response to SLIT

We then sought to identify the key differences between AR patients responding and not responding to SLIT. Among the 27 SLIT-treated patients, 12 who had received treatment for longer than 6 months were included in the response analysis and classified as 6 responders and 6 non-responders according to the predefined symptom-based criteria (Figure 1; see the relevant Materials and Methods section). The VAS scores of the responder and non-responder groups were 9.67 ± 3.94 and 18 ± 5.26, respectively (mean ± SD, *P* < 0.05), and the demographic features were comparable (Table 2).

**Figure 3:**
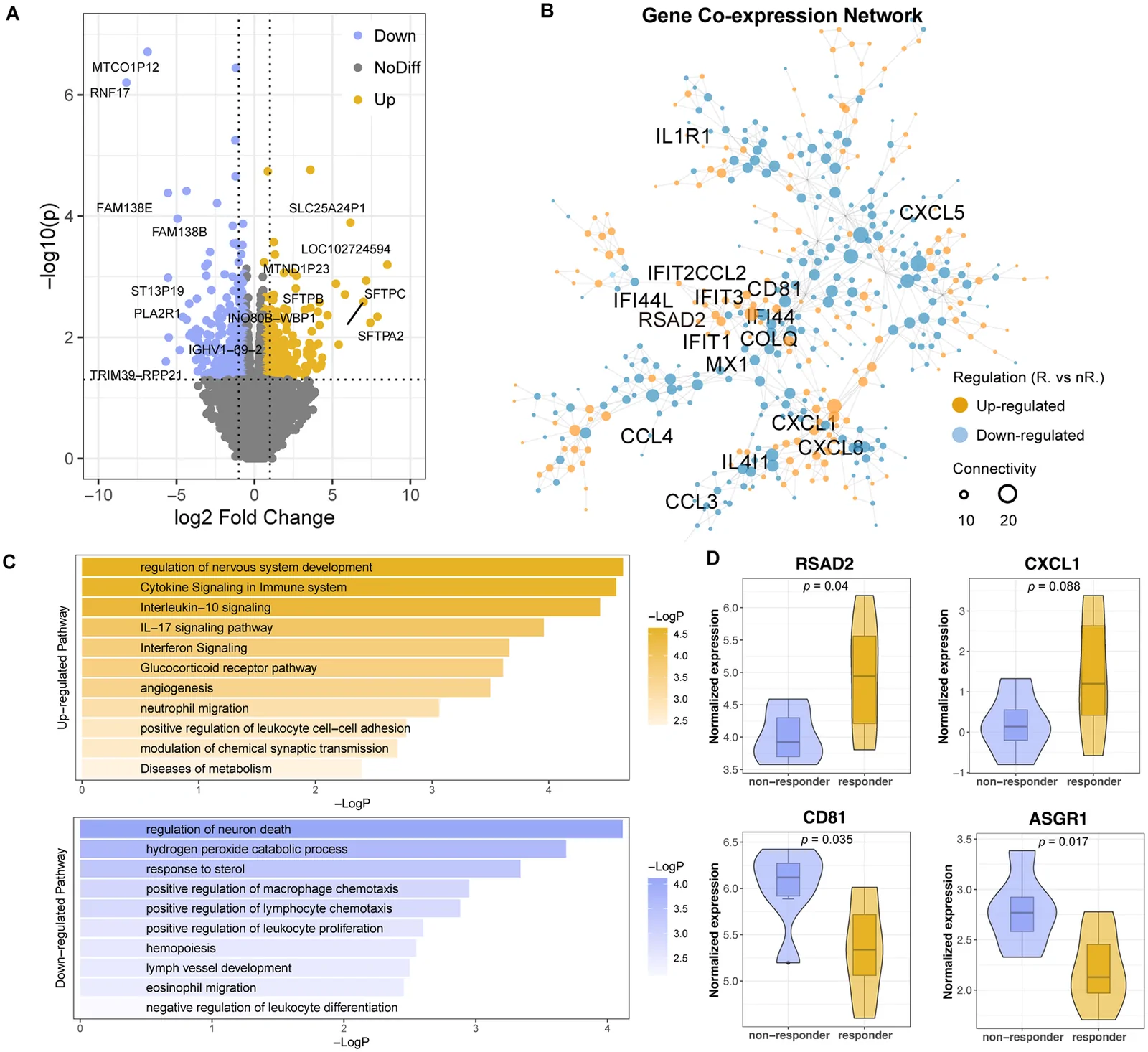
Genes associated with SLIT response **(A)** volcano plot showing the differentially expressed genes (DEGs) between sublingual immunotherapy responders and non-responders (log2 fold change > 0.58, FDR < 0.05); the top genes are labeled. **(B)** Network diagram of the DEGs’ co-expression analysis, with the selected hub genes labeled. **(C)** The top enriched pathways of genes up- and down-regulated in the responder group vs. the non-responder group. **(D)** Violin plot showing the expression of selected hub genes. (two-tailed t-test, showing *P*-value).

The DEG analysis identified 257 up-regulated and 349 down-regulated genes in the responders (Figure 3A). Similarly, we used gene co-expression network to identify the gene expression signatures of SLIT responders and non-responders (Figure 3B). The network analysis revealed genes associated with neutrophil migration (CXCL1, CXCL5, CXCL8), lymphocyte regulation (CD81), and interferon signaling (RSAD2, MX1, IFI44L).

### Multiple immunoregulatory pathways are up-regulated in SLIT responders

Gene sets enrichment analysis revealed that the interferon signaling (*HLA-DQA1, HLA-DRB5, IFIT1, MX1, RSAD2*), IL-10 signaling (*IL1R1, PTGS2*) and IL-17 signaling (*CXCL1, FOSB, CCL2*) pathways were up-regulated in the responder group. By contrast, the leukocyte proliferation (*BCL2L1, CD81, TNFRSF4*) and chemotaxis (*THBS1, IL34, CMKLR1*) pathways were significantly elevated in the non-responder group (Figure 3C). Notably, genes associated with neutrophil migration (*EMP2, CXCL5, CXCL8*) and eosinophil migration (*CCL3, CCL4, XCL1*) were respectively up-regulated in the responder and non-responder groups, suggesting a shift in granulocyte infiltration patterns had occurred. Additionally, the regulatory pathway of neural system development (*CDH1, EGR2, NTRK3*) was up-regulated in the responders, whereas the pathway for neuronal death (*GHR, TYRO3, FZD1*) was up-regulated in the non-responder group. Expression violin plot further validate the different expression of selected genes (Figure 3D).

### NK-cell-associated signals are linked to SLIT efficacy

**Figure 4:**
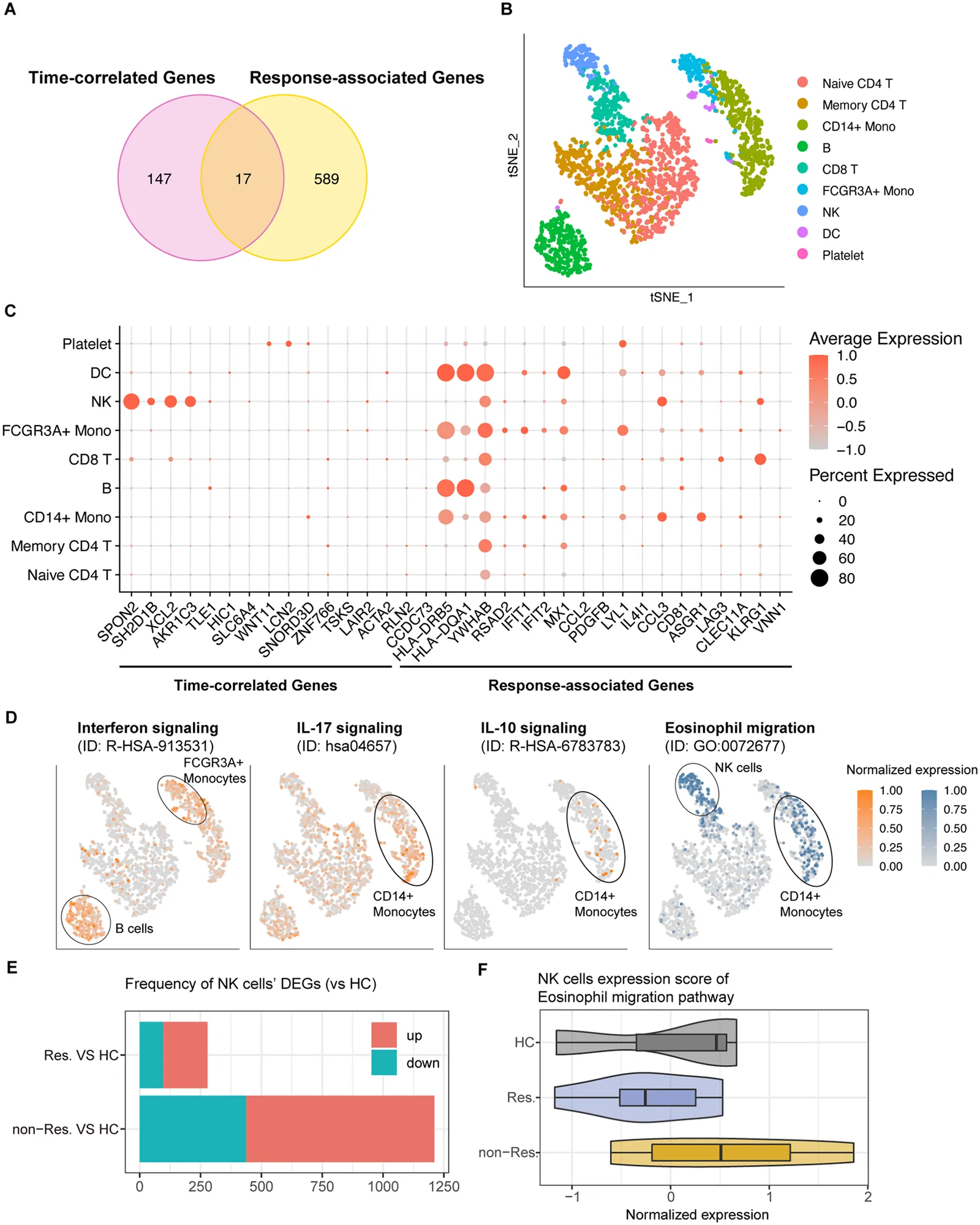
Cellular expression map of SLIT-associated genes and pathways **(A)** venn plot showing the numbers of SLIT time-correlated genes and response-associated genes and their overlap. **(B)** tSNE plot showing the clustering and annotation result of the PBMC single cell transcriptomic data. **(C)** Dot plot showing the expression of selected genes across all the cell types evaluated. **(D)** Feature plot showing the gene set scores of up-regulated pathways in responders (orange) and non-responders (blue). **(E)** Bar plot showing the DEGs frequencies of natural killer (NK) cells of 2 comparison group, responders vs. healthy control and non-responder vs. healthy control (fold change > 2, FDR < 0.05). **(F)** Violin plot showing the gene set score of the “eosinophil migration” pathway (GO:0072677) in the NK cells of healthy controls (grey), responders (orange), and non-responders (blue). Panels E and F provide exploratory deconvolution-based support for NK-cell-associated differences related to SLIT response.

Having identified genes and pathways associated with the duration of and response to SLIT. A couple of time-correlated genes were also identified as differentially expressed genes, such as SPON2 and SLC4A1 (Figure 4A). The complete list is indicated in Supplementary Table S3.

We wished to further elucidate which cell types were expressing these genes. To this end, we re-analyzed a publicly available PBMC single-cell RNA-seq dataset, and nine cell types were recovered, including naive CD4+ T cells, memory CD4+ T cells, CD8+ T cells, B cells, CD14 + monocytes, FCGR3A + monocytes, DCs, NK cells, and platelets (Figure 4B). The expression of selected genes (Figure 4C) and pathways (Figure 4D) are shown. We found that the Interferon signaling pathway (R-HSA-913531) showed relatively higher expression scores in FCGR3A + monocytes and B cells, while CD14 + monocytes also showed relatively high IL-10 signaling (R-HSA-6783783) and IL-17 signaling pathway (hsa04657) scores. The eosinophil migration pathway (GO:0072677), which was down-regulated in the responders, also showed relatively high expression in NK cells and CD14 + monocytes.

We noticed that NK cells expressed T-cell-regulatory-associated genes such as SPON2, SH2D1B, and AKR1C3, as well as XCL2, a gene included in eosinophil-migration-related signatures. Hence, we additionally implemented a deconvolution algorithm in our data to compare the NK-cell expression profiles among responders, non-responders, and HCs (see the relevant Materials and Methods section). Because larger DEG counts suggest greater transcriptional differences between 2 groups and vice versa, the comparison of DEG numbers suggested that the NK-cell profiles of responders were more similar to those of HCs than were those of non-responders (Figure 4E). Moreover, the “eosinophil migration” pathway score was similar in NK cells from HCs and responders, but higher in those from non-responders (Figure 4F). Taken together, these findings suggest that NK-cell-associated transcriptional differences, including eosinophil-migration-related signals, may be linked to SLIT efficacy.

## Discussion

Time-correlated genes and response-associated genes of SLIT can provide valuable insights into the biological effects of the treatment. Our study primarily utilized high-throughput methodologies, examining from the aforementioned two perspectives, delineating a set of potential biological targets. We employed bioinformatics methods to preliminarily explore the functions and cellular expressions of these genes, laying the groundwork for subsequent research.

Our results first found that the expression levels of several genes were significantly associated with SLIT duration in patients' PBMCs. Firstly, several markers associated with innate-immune-response-mediated T cell suppression were identified. For example, SH2D1B (EAT-2), which belongs to the signaling lymphocytic activation molecule (SLAM) family and is mostly expressed by antigen presenting cells, works together with the SLAM-associated protein (SAP) to regulate T cell function (24, 25). Moreover, there was ARG1, which encodes arginase 1 and was primarily expressed by myeloid cells. Arginase 1 inhibits T cell proliferation and cytokine secretion by competing with nitric oxide synthase for its arginine substrate (26, 27). Additionally, the loss of TLE1 was shown to increase the activity of the proinflammatory NF-*κ*B pathway and promote the systemic inflammatory response *in vivo* (28). Thus, these genes indicate that the regulation of T cell function gradually occurred as the treatment proceed.

To date, no robust biomarkers of SLIT-induced symptom relief have been found, since studies of IgE and IgG4 levels have yielded conflicting results (29, 30). Interestingly, our transcriptomic results showed that the expression of SLC6A4, which encodes the 5-HT transporter, increased with SLIT duration, and qPCR in the available PBMC samples supported this positive association. Elevated 5-HT concentration, both locally and systematically, contributes to primary AR symptoms, such as nasal itching and sneezing (31). Meanwhile, the expression of SLC6A4 lowers 5-HT concentrations in the synaptic cleft by re-uptake (32). Thus, increased SLC6A4 expression may indicate the improvement of AR symptoms during SLIT. In the same qPCR set, SH2D1B and ARG1 also showed positive but non-significant trends, further supporting the overall reliability of the duration-associated transcriptomic signal while still warranting validation in larger cohorts.

We also explored signatures associated with the efficacy of SLIT. Our results showed that the responder group up-regulated IL-10 signaling compared to non-responders, in line with previous findings (33, 34). Interestingly, we also identified a difference in the pattern of granulocyte migration between the SLIT responders and non-responders. Previous studies have shown that the numbers of eosinophils in the nasal mucosa and blood were increased in patients with AR vs. HCs, which was associated with multiple pathological mechanisms (1, 35). Moreover, the ratio of eosinophils to neutrophils positively correlates with AR severity (36). AIT was also shown to alleviate eosinophil infiltration in the airways of mice used as allergy models (37).

In the present study, we further used single-cell transcriptomic information to refine the findings at the cellular level. Among the nine PBMC cell clusters, NK cells showed relatively high expression of eosinophil-migration-related signatures. The NK-cell profiles of responders and HCs appeared more similar, whereas those of non-responders appeared more distinct. The deconvolution-based analysis further suggested that NK cells from SLIT non-responders had higher “eosinophil migration” pathway scores than those from responders and HCs. These observations suggest that altered eosinophil-migration-related signals in NK cells may be ssociated with SLIT efficacy, but this interpretation remains hypothesis-generating.

In addition to patterns related to granulocyte chemotaxis, NK cells also expressed several hub genes correlated with SLIT treatment duration, including SPON2, SH2D1B, and AKR1C3, suggesting a possible link to T-cell-associated immune modulation. Literature has provided some pertinent insights. For instance, Deniz et al. demonstrated that NK cells could directly impact the balance between type 1 and type 2 inflammation by altering their functional identity (38). A distinct subset of NK cells, termed regulatory NK cells (NKregs), is found to be diminished in AR patients compared with HCs and is recognized for its capability to release transforming growth factor (TGF)-β and IL-10, implying a potential role for these cells in mitigating allergic inflammation (39). These prior observations support the biological plausibility of NK-cell involvement, but the present transcriptomic results do not establish a direct mechanism.

Taken together, our results preliminarily found a set of genes associated with SLIT efficacy. However, as an exploratory transcriptomic study, this work also has several limitations, including the cross-sectional design, the small number of healthy controls and the resulting limited statistical power for HC-related comparisons, the symptom-based evaluation of treatment efficacy without routine nasal provocation testing, the use of a healthy-donor public PBMC dataset as the single-cell reference for exploratory deconvolution, and limited experimental validation beyond the qPCR analysis of three duration-associated genes. Further research using longitudinal study designs, larger and better-balanced cohorts, and disease-matched single-cell datasets will be needed to validate and refine these findings. Accordingly, the present findings, particularly the HC-related comparisons and single-cell/NK-cell-related inferences, should be interpreted as supportive and exploratory rather than definitive.

## Conclusion

This study revealed genes associated with SLIT duration and efficacy and preliminarily explore their functional characteristics using bioinformatic tools. These results can provide useful reference for further research to experimentally validate and develop biomarkers for SLIT.

## Data Availability

The datasets presented in this study can be found in online repositories. The names of the repository/repositories and accession number(s) can be found in the article/Supplementary Material. The RNA-seq dataset is available in the Sequence Read Archive under accession PRJNA808860.

## References

[1] Chen Y Yang M Deng J Wang K Shi J Sun Y. Elevated levels of activated and pathogenic eosinophils characterize moderate-severe house dust Mite allergic rhinitis. J Immunol Res. (2020) 2020:8085615. 10.1155/2020/8085615 32855977 PMC7443015

[2] Meltzer EO Blaiss MS Naclerio RM Stoloff SW Derebery MJ Nelson HS. Burden of allergic rhinitis: allergies in America, Latin America, and Asia-Pacific adult surveys. Allergy Asthma Proc. (2012) 33(Suppl 1):S113–41. 10.2500/aap.2012.33.3603 22981425

[3] Zhang L Han D Huang D Wu Y Dong Z Xu G. Prevalence of self-reported allergic rhinitis in eleven major cities in China. Int Arch Allergy Appl Immunol. (2009) 149:47–57. 10.1159/000176306 19033732

[4] Pfaar O Lou H Zhang Y Klimek L Zhang L. Recent developments and highlights in allergen immunotherapy. Allergy. (2018) 73:2274–89. 10.1111/all.13652 30372537

[5] Lee J-E Choi Y-S Kim M-S Han DH Rhee C-S Lee CH. Efficacy of sublingual immunotherapy with house dust mite extract in polyallergen sensitized patients with allergic rhinitis. Ann Allergy Asthma Immunol. (2011) 107:79–84. 10.1016/j.anai.2011.03.012 21704889

[6] Liu Z Lu H Feng X Hu L Wang J Yu H. Predictive methods for efficacy of house dust mite subcutaneous immunotherapy in allergic rhinitis patients: a prospective study in a Chinese population. Int Forum Allergy Rhinol. (2020) 10:314–9. 10.1002/alr.22508 31869861

[7] Zhu K Xia C Chen J Yu C Gao T Yan J. Serum soluble ST2 correlated with symptom severity and clinical response of sublingual immunotherapy for house dust Mite-induced allergic rhinitis patients. Mediat Inflamm. (2021) 2021:5576596. 10.1155/2021/5576596 PMC8181096 34194284

[8] Incorvaia C Riario-Sforza GG Incorvaia S Frati F. Sublingual immunotherapy in allergic asthma: current evidence and needs to meet. Ann Thorac Med. (2010) 5:128–32. 10.4103/1817-1737.65038 20835305 PMC2930649

[9] Akdis CA Akdis M. Advances in allergen immunotherapy: aiming for complete tolerance to allergens. Sci Transl Med. (2015) 7:280ps6. 10.1126/scitranslmed.aaa7390 25810310

[10] Durham SR Emminger W Kapp A De Monchy JGR Rak S Scadding GK. SQ-standardized sublingual grass immunotherapy: confirmation of disease modification 2 years after 3 years of treatment in a randomized trial. J Allergy Clin Immunol. (2012) 129:717–725.e5. 10.1016/j.jaci.2011.12.973 22285278

[11] Huoman J Papapavlou G Pap A Alm J Nilsson LJ Jenmalm MC. Sublingual immunotherapy alters salivary IgA and systemic immune mediators in timothy allergic children. Pediatr Allergy Immunol. (2019) 30:522–30. 10.1111/pai.13047 30803044

[12] Głobińska A Boonpiyathad T Satitsuksanoa P Kleuskens M Van De Veen W Sokolowska M. Mechanisms of allergen-specific immunotherapy: diverse mechanisms of immune tolerance to allergens. Ann Allergy Asthma Immunol. (2018) 121:306–12. 10.1016/j.anai.2018.06.026 29966703

[13] Brożek JL Bousquet J Agache I Agarwal A Bachert C Bosnic-Anticevich S. Allergic rhinitis and its impact on asthma (ARIA) guidelines-2016 revision. J Allergy Clin Immunol. (2017) 140:950–8. 10.1016/j.jaci.2017.03.050 28602936

[14] Di Lorenzo G Mansueto P Pacor ML Rizzo M Castello F Martinelli N. Evaluation of serum s-IgE/total IgE ratio in predicting clinical response to allergen-specific immunotherapy. J Allergy Clin Immunol. (2009) 123:1103–1110.e4. 10.1016/j.jaci.2009.02.012 19356792

[15] Liao Y Smyth GK Shi W. Featurecounts: an efficient general purpose program for assigning sequence reads to genomic features. Bioinformatics. (2014) 30:923–30. 10.1093/bioinformatics/btt656 24227677

[16] Pertea M Kim D Pertea GM Leek JT Salzberg SL. Transcript-level expression analysis of RNA-Seq experiments with HISAT, StringTie and ballgown. Nat Protoc. (2016) 11:1650–67. 10.1038/nprot.2016.095 27560171 PMC5032908

[17] Song WM Zhang B. Multiscale embedded gene co-expression network analysis. PLoS Comput Biol. (2015) 11:e1004574. 10.1371/journal.pcbi.1004574 26618778 PMC4664553

[18] Shannon P Markiel A Ozier O Baliga NS Wang JT Ramage D. Cytoscape: a software environment for integrated models of biomolecular interaction networks. Genome Res. (2003) 13:2498–504. 10.1101/gr.1239303 14597658 PMC403769

[19] Robinson MD McCarthy DJ Smyth GK. Edger: a bioconductor package for differential expression analysis of digital gene expression data. Bioinformatics. (2010) 26:139–40. 10.1093/bioinformatics/btp616 19910308 PMC2796818

[20] Zhou Y Zhou B Pache L Chang M Khodabakhshi AH Tanaseichuk O. Metascape provides a biologist-oriented resource for the analysis of systems-level datasets. Nat Commun. (2019) 10:1523. 10.1038/s41467-019-09234-6 30944313 PMC6447622

[21] Hänzelmann S Castelo R Guinney J. GSVA: gene set variation analysis for microarray and RNA-Seq data. BMC Bioinformatics. (2013) 14:7. 10.1186/1471-2105-14-7 23323831 PMC3618321

[22] Hao Y Hao S Andersen-Nissen E Mauck WM Zheng S Butler A. Integrated analysis of multimodal single-cell data. Cell. (2021) 184:3573–87.e29. 10.1016/j.cell.2021.04.048 34062119 PMC8238499

[23] Chu T Wang Z Pe’er D Danko CG. Cell type and gene expression deconvolution with BayesPrism enables Bayesian integrative analysis across bulk and single-cell RNA sequencing in oncology. Nat Cancer. (2022) 3:505–17. 10.1038/s43018-022-00356-3 35469013 PMC9046084

[24] Ma CS Nichols KE Tangye SG. Regulation of cellular and humoral immune responses by the SLAM and SAP families of molecules. Annu Rev Immunol. (2007) 25:337–79. 10.1146/annurev.immunol.25.022106.141651 17201683

[25] Detre C Keszei M Romero X Tsokos GC Terhorst C. SLAM Family receptors and the SLAM-associated protein (SAP) modulate T cell functions. Semin Immunopathol. (2010) 32:157–71. 10.1007/s00281-009-0193-0 20146065 PMC2868096

[26] Steggerda SM Bennett MK Chen J Emberley E Huang T Janes JR. Inhibition of arginase by CB-1158 blocks myeloid cell-mediated immune suppression in the tumor microenvironment. J Immunother Cancer. (2017) 5:101. 10.1186/s40425-017-0308-4 29254508 PMC5735564

[27] Bronte V Zanovello P. Regulation of immune responses by L-arginine metabolism. Nat Rev Immunol. (2005) 5:641–54. 10.1038/nri1668 16056256

[28] Chen W Zheng D Mou T Pu J Dai J Huang Z. Tle1 attenuates hepatic ischemia/reperfusion injury by suppressing NOD2/NF-*κ*B signaling. Biosci, Biotechnol, Biochem. (2020) 84:1176–82. 10.1080/09168451.2020.1735928 32114961

[29] Shamji MH Layhadi JA Sharif H Penagos M Durham SR. Immunological responses and biomarkers for allergen-specific immunotherapy against inhaled allergens. J Allergy Clin Immunol Pract. (2021) 9:1769–78. 10.1016/j.jaip.2021.03.029 33781958

[30] Ciprandi G Fenoglio D Cirillo I Tosca MA La Rosa M Licari A. Sublingual immunotherapy: an update on immunologic and functional effects. Allergy Asthma Proc. (2007) 28:40–3. 10.2500/aap.2007.28.2974 17390756

[31] Yang G Wu G Yao W Guan L Geng X Liu J. 5-HT Is associated with the dysfunction of regulating T cells in patients with allergic rhinitis. Clinical Immunology (Orlando, Fla). (2022) 243:109101. 10.1016/j.clim.2022.109101 36029976

[32] Gelernter J. SLC6A4 Polymorphism, population genetics, and psychiatric traits. Hum Genet. (2014) 133:459–61. 10.1007/s00439-013-1412-2 24385047 PMC3992709

[33] Francis JN Till SJ Durham SR. Induction of IL-10 + CD4 + CD25+ T cells by grass pollen immunotherapy. J Allergy Clin Immunol. (2003) 111:1255–61. 10.1067/mai.2003.1570 12789226

[34] Bohle B Kinaciyan T Gerstmayr M Radakovics A Jahn-Schmid B Ebner C. Sublingual immunotherapy induces IL-10-producing T regulatory cells, allergen-specific T-cell tolerance, and immune deviation. J Allergy Clin Immunol. (2007) 120:707–13. 10.1016/j.jaci.2007.06.013 17681368

[35] Amin K Issa SM Ali KM Aziz MI Hama Amieen HM Bystrom J. Evidence for eosinophil and IL-17 mediated inflammation in allergic rhinitis. Clinical and Molecular Allergy: CMA. (2020) 18:6. 10.1186/s12948-020-00117-6 32280308 PMC7129325

[36] Kant A Terzioğlu K. Association of severity of allergic rhinitis with neutrophil-to-lymphocyte, eosinophil-to-neutrophil, and eosinophil-to-lymphocyte ratios in adults. Allergol Immunopathol (Madr). (2021) 49:94–9. 10.15586/aei.v49i5.204 34476928

[37] Aguilar-Pimentel A Graessel A Alessandrini F Fuchs H Gailus-Durner V Hrabě de Angelis M. Improved efficacy of allergen-specific immunotherapy by JAK inhibition in a murine model of allergic asthma. PLoS One. (2017) 12:e0178563. 10.1371/journal.pone.0178563 28570653 PMC5453633

[38] Deniz G Van De Veen W Akdis M. Natural killer cells in patients with allergic diseases. J Allergy Clin Immunol. (2013) 132:527–35. 10.1016/j.jaci.2013.07.030 23993354

[39] Boonpiyathad T Lao-Araya M Chiewchalermsri C Sangkanjanavanich S Morita H. Allergic rhinitis: what do we know about allergen-specific immunotherapy? Front Allergy. (2021) 2:747323. 10.3389/falgy.2021.747323 35387059 PMC8974870

